# Early myocardial damage (EMD) and valvular insufficiency result in impaired cardiac function after multiple trauma in pigs

**DOI:** 10.1038/s41598-020-80409-8

**Published:** 2021-01-13

**Authors:** Birte Weber, Ina Lackner, Meike Baur, Florian Gebhard, Roman Pfeifer, Paolo Cinelli, Sascha Halvachizadeh, Michel Teuben, Hans-Christoph Pape, Armin Imhof, Miriam Lipiski, Nikola Cesarovic, Miriam Kalbitz

**Affiliations:** 1 Department of Traumatology, Hand, Plastic and Reconstructive Surgery, Center of Surgery, University of Ulm Medical School, Albert-Einstein-Allee 23, 89081 Ulm, Germany; 2 Department of Trauma, University Hospital of Zurich, Zurich, Switzerland; 3 Department of Internal Medicine II-Cardiology, University of Ulm Medical Centre, Ulm, Germany; 4 Department of Surgical Research, University Hospital of Zurich, Zurich, Switzerland; 5 Department of Health Sciences, Translational Cardiovascular Technologies, Swiss Federal Institute of Technology, Zurich, Switzerland

**Keywords:** Medical research, Translational research

## Abstract

One third of multiple trauma patients present abnormal echocardiographic (ECHO) findings. Therefore, ECHO diagnostic after trauma is indicated in case of hemodynamic instability, shock, after chest trauma and after cardiac arrest. 20 male pigs underwent multiple trauma. Blood samples were collected 4 and 6 h after trauma and concentrations of heart-type fatty acid binding protein (HFABP) as a biomarker for EMD were measured. Myocardial damage was evaluated by scoring Hematoxylin–Eosin stained sections. At baseline, 3 and 6 h after trauma, transesophageal ECHO (TOE) was performed, invasive arterial and left ventricular blood pressure were measured to evaluate the cardiac function after multiple trauma. Systemic HFABP concentrations were elevated, furthermore heart injury score in multiple trauma animals was increased determining EMD. A significant decrease of blood pressure in combination with a consecutive rise of heart frequency was observed. Ongoing depression of mean arterial pressure and diastolic blood pressure were accompanied by changes in ECHO-parameters indicating diastolic and systolic dysfunction. Furthermore, a valvular dysfunction was detected. In this study complex myocardial and valvular impairment after multiple trauma in pigs has been observed. Therefore, detection of EMD and progressive valvular dysfunction might be crucial and therapeutically relevant.

## Introduction

In almost one third of multiple trauma patients hospitalization is due to chest trauma, which is responsible for 20–25% of multiple trauma related deaths^1^. Abnormal echocardiographic (ECHO) findings were detected in 31% of multiple trauma patients, which included 20% patients with ventricular wall motion abnormalities^2^. Therefore, ECHO plays an important role in emergency medicine as a non-invasive method that is universally available and allows therapeutic relevant decisions^3^. The indication for echocardiography after trauma is given in cases of hemodynamic instability, shock, chest trauma, as well as after cardiac arrest with cardiopulmonary resuscitation^3^. In trauma patients, arrhythmias, cardiac murmurs or ongoing hypotension alerts physicians to suspect a cardiovascular trauma, which potentially life-threatening^4–7^. Because of the anterior location of the heart in the thorax, the right ventricle (17–32%) and the atria (8–65%) are the most commonly injured parts of the heart in humans^8,9^. In pigs a reduction of the ejection fraction (EF) as well as an impaired shortening fraction was observed 1.5 h after multiple trauma, which was reported to be reversible after 24 h^10^. During thoracic trauma the heart is susceptible to compression within the bony structures of the thorax, whereas abdominal compression can lead to a rapid increase in blood flow to the heart, which could be responsible for a ventricular rupture, caused by the enormous increase of intracardial pressure^11,12^. Furthermore, after car accidents single case reports of severe valvular regurgitations, rupture of the anterolateral papillary muscle or atypical septum defects were described^13,14^.

Because of the high quality of transesophageal ECHO (TOE) measurements^11^, in the present study we used this investigation method to evaluate early myocardial damage (EMD) after multiple trauma in pigs. ECHO after trauma is feasible to detect cardiac contusion, because heart contusion is associated with oedematous changes of the myocardium, which lead to an increase of echogenity and thickness^15^. Additionally, injuries of the coronary vessels can be translated in regional wall motion abnormalities of the ventricle detectable by ECHO^15^.

In case of blunt chest trauma, troponin I and T were reported as the featured indicator of cardiac damage^16^. Increased systemic troponin levels after multiple trauma in patients have been associated with high injury severity score (ISS), poor outcome and the necessity to administrate additional fluids as well as catecholamines^2,17^. In critically ill patients, elevated cardiac troponin T levels were associated with high in-hospital mortality but did not correlate with long-term survival^18^. Especially the combination of cardiac damage markers such as troponin or HFABP with ECHO findings might ensure an early detection of cardiac complications after trauma^19,20^.

Therefore, the aim of this study was to conduct a detailed analysis of cardiac function after multiple trauma in pigs in combination with local and systemic detection of cardiac damage. We hypothesized that multiple trauma in pigs leads to EMD with systolic and diastolic cardiac dysfunction and impaired valvular function.

## Results

**Figure 1:**
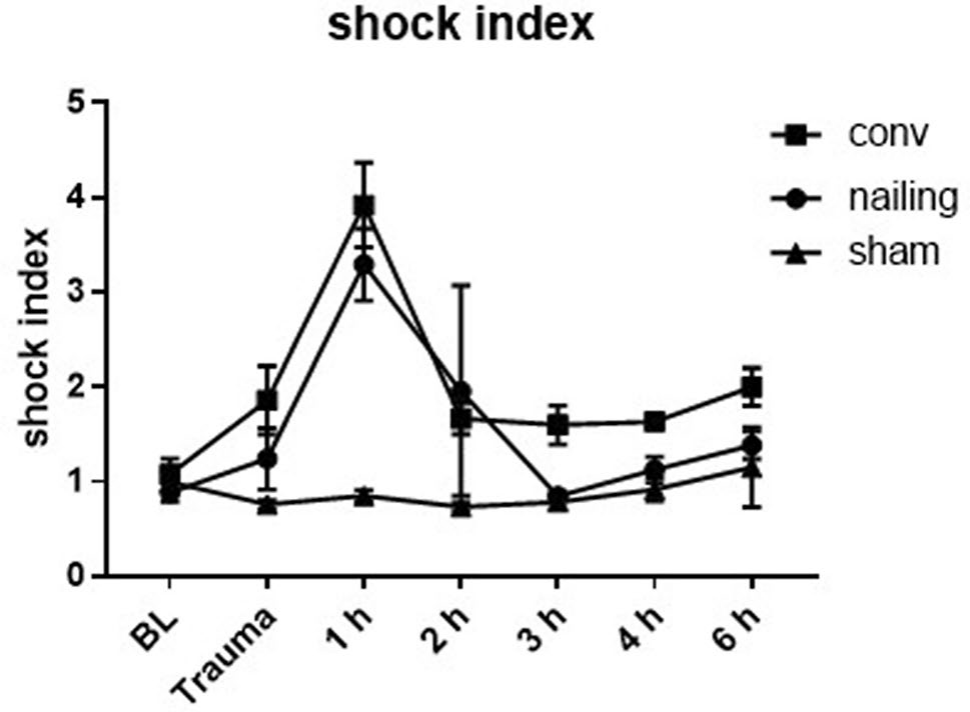
Shock index**.** Shock index calculated as heart rate/systolic blood pressure. Combined multiple trauma group n = 10, femoral nailing n = 5, conventional reaming n = 5, sham = 5. *p < 0.05.

In order to demonstrate hemodynamic alterations after multiple trauma in pigs, we analyzed the heart rate and the blood pressure after trauma over 6 h. The shock index was increased during the trauma period (Fig. 1) in both groups, whereas the conventional reaming group presented higher values of the shock index during the observation period. 1 h after trauma the shock index was significantly increased in both trauma groups compared to sham.

**Figure 2:**
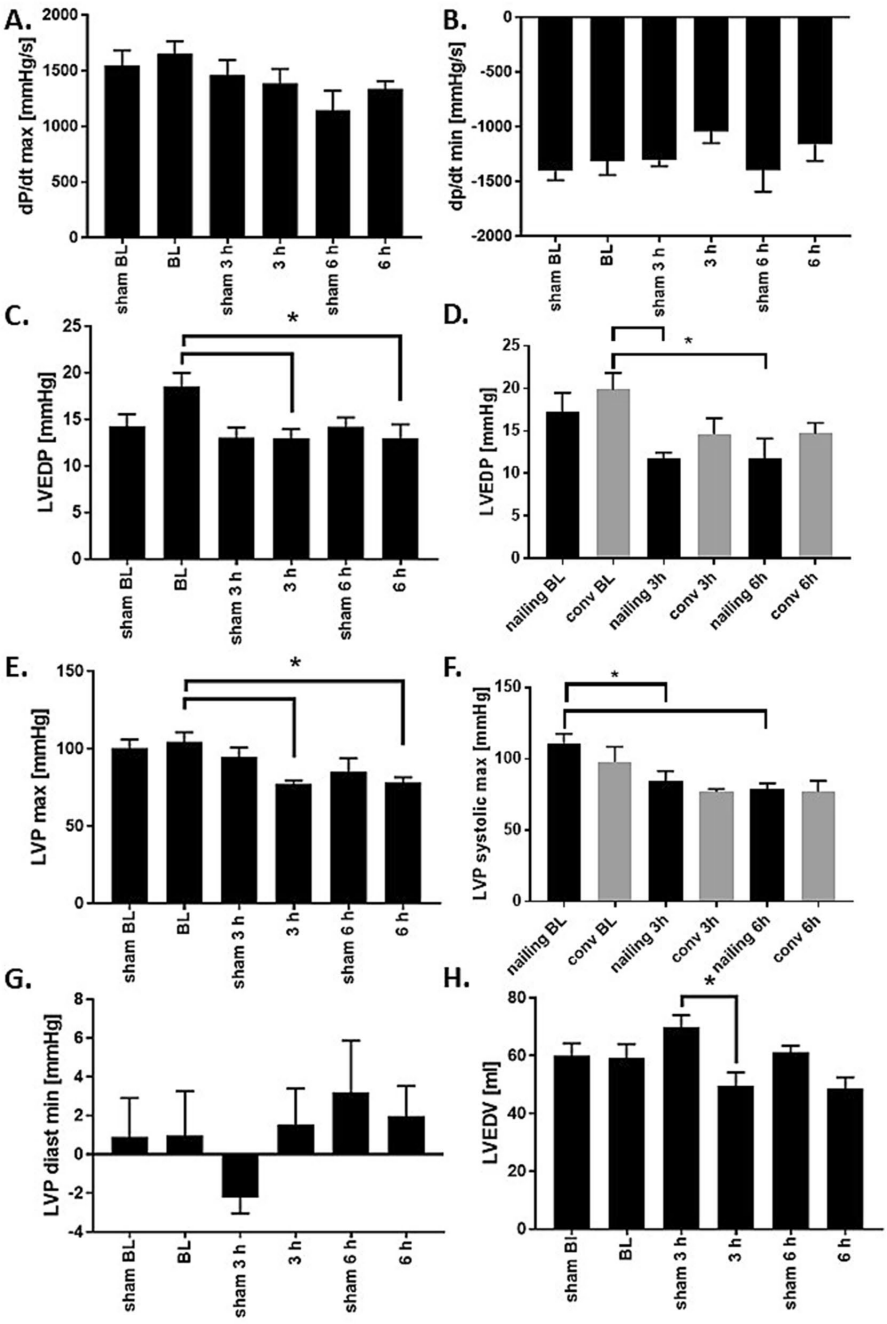
Cardiac function after multiple trauma—blood pressure. (**A**) dp/dt max in mmHg/s at baseline (BL), 3 and 6 h after multiple trauma (femur fracture, liver laceration, chest trauma and hemorrhagic shock) in pigs, presented as a combined trauma group consisting of femoral nailing and conventional reaming as treatment. (**B**) dp/dt min in mmHg/s at baseline (BL), 3 and 6 h after trauma in pigs. (**C**) Left ventricular end diastolic pressure (LVEDP) in mmHg at BL, 3 and 6 h after multiple trauma in pigs, p* < 0.05 compared to baseline. (**D**) Subgroup analysis of left ventricular end diastolic pressure in mmHg in animals with femoral nailing (nailing, black) or conventional reaming (conv, gray) at BL, 3 h and 6 h after multiple trauma, *p > 0.05 compared to baseline. (**E**) Maximal left ventricular pressure (LVP max) in mmHg at BL, 3 and 6 h after multiple trauma, *p < 0.05. (**F**) Subgroup analysis of maximal left ventricular pressure (LVP max) in mmHg in animals with femoral nailing (nailing, black) or conventional reaming (conv, gray) at BL, 3 and 6 h after multiple trauma, *p > 0.05 compared to baseline. (**G**) Minimal left ventricular pressure (LVP min) in mmHg at BL, 3 and 6 h after multiple trauma, *p < 0.05. (**H**) Left ventricular enddiastolic volume in ml. Combined multiple trauma group n = 10, femoral nailing n = 5, conventional reaming n = 5, sham n = 5.

To describe early myocardial damage in detail, we analyzed parameters of the continuous blood pressure measurement. In the first 6 h after trauma, the contractility parameter dp/dt max as well as the dp/dt min were not influenced by multiple trauma (Fig. 2A,B). However, we measured a decrease of the left ventricular end diastolic pressure (LVEDP) in the combined multiple trauma group after 3 and 6 h (Fig. 2C). Subgroup analysis demonstrated differences in the left ventricular end diastolic pressure between conventional reaming and femoral nailing (Fig. 2D). To get a complete picture of the overall pressure relationships in the heart, we measured the maximal left ventricular pressure (LVPmax) as well as the minimal left ventricular pressure (LVPmin). In the combined multiple trauma group a reduction of the LVPmax was observed after 3 and 6 h (Fig. 2E). This observation was also made in the subgroup analysis of the femoral nailing group (Fig. 2F). LVPmin was not influenced by the multiple trauma with haemorrhage in all conducted groups (Fig. 2G). We did not observe changes in the left ventricular enddiastolic volume compared to baseline. (Fig. 2H), but a significantly reduced value 3 h after trauma compared to sham treated animals.

**Figure 3:**
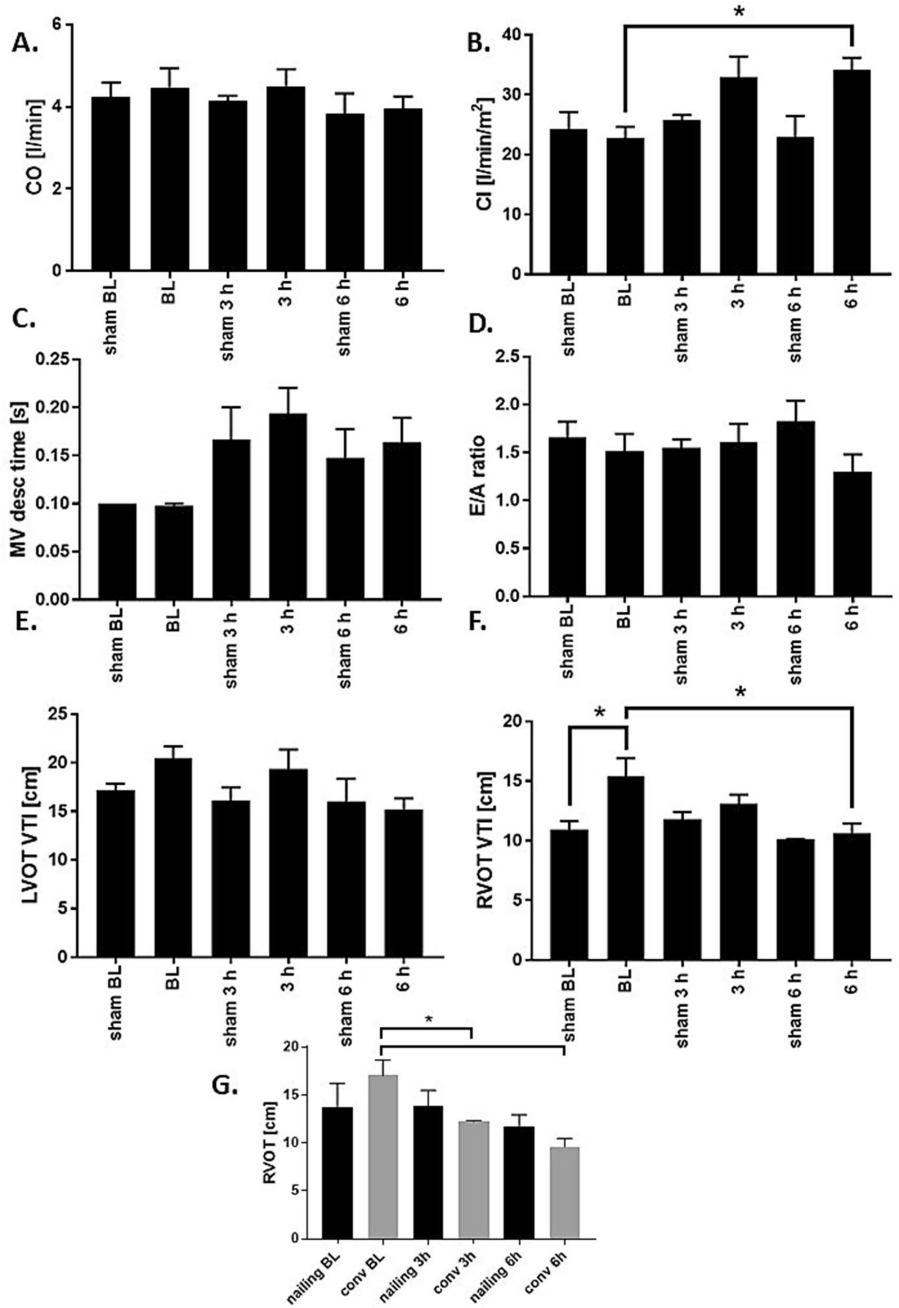
Cardiac function—echocardiography—parameters. (**A**) Cardiac output (CO) in l/min at baseline (BL), 3 and 6 h after multiple trauma in pigs (femur fracture, liver laceration, chest trauma and hemorrhagic shock) presented as a combined trauma group containing femoral nailing and conventional reaming as therapeutic treatment. (**B**) Contractility index (CI) in l/min/m^2^ measured at BL, 3 and 6 h after multiple trauma, *p < 0.05 compared to baseline. (**C**) Mitral valve deceleration time (MV desc time) in sec detected at BL, 3 and 6 h after trauma in pigs, *p < 0.05 compared to baseline. (**D**) Ratio of E wave and A wave (E/A) measured at BL, 3 and 6 h after multiple trauma with hemorrhagic shock. (**E**) Left ventricular tract velocity time integral (LVOT VTI) in cm at BL, 3 and 6 h after trauma, *p = 0.06 compared to baseline. (**F**) Right ventricular outflow tract velocity time integral (RVOT VTI) in cm measured at BL, 3 and 6 h after multiple trauma, *p < 0.05 compared to baseline. (**G**) Subgroup analysis of right ventricular outflow tract velocity time integral (RVOT VTI) in cm at BL, 3 and 6 h after trauma and either conventional reaming (conv, gray) or femoral nailing (nailing, black), * p < 0.05 compared to baseline. Combined multiple trauma group n = 10, femoral nailing n = 5, conventional reaming n = 5, sham n = 5.

As standard parameters of systolic cardiac function we measured the cardiac output and the ejection fraction after multiple trauma by transoesophageal echocardiography. We did not detect changes in cardiac output the combined multiple trauma group (Fig. 3A). However, we found an increase of the contractility index 3 and 6 h after trauma in the multiple injured animals (Fig. 3B). As a parameter of diastolic dysfunction of the left ventricle, we measured the mitral valve deceleration time and detected a significant increase in the combined trauma group compared to baseline measurements (Fig. 3C). Baseline values and increased post-traumatic values were measured in the normal range of deceleration (140–240 ms). To determine the diastolic filling of the left ventricle over the mitral valve, we detected the E/A ratio after 3 and 6 h, but did not find any significant changes in the ratio of E wave to A wave (Fig. 3D.). LVOT- and RVOT-VTI were reduced after 6 h in the combined multiple trauma group (Fig. 3E,F). Subgroup analysis presented a reduction of the RVOT-VTI after 3 h in the multiple trauma animals with conventional reaming compared to baseline values (Fig. 3G). Additional ECHO measurements are presented in the supplemental Fig. 1 (Fig. S1).

**Figure 4:**
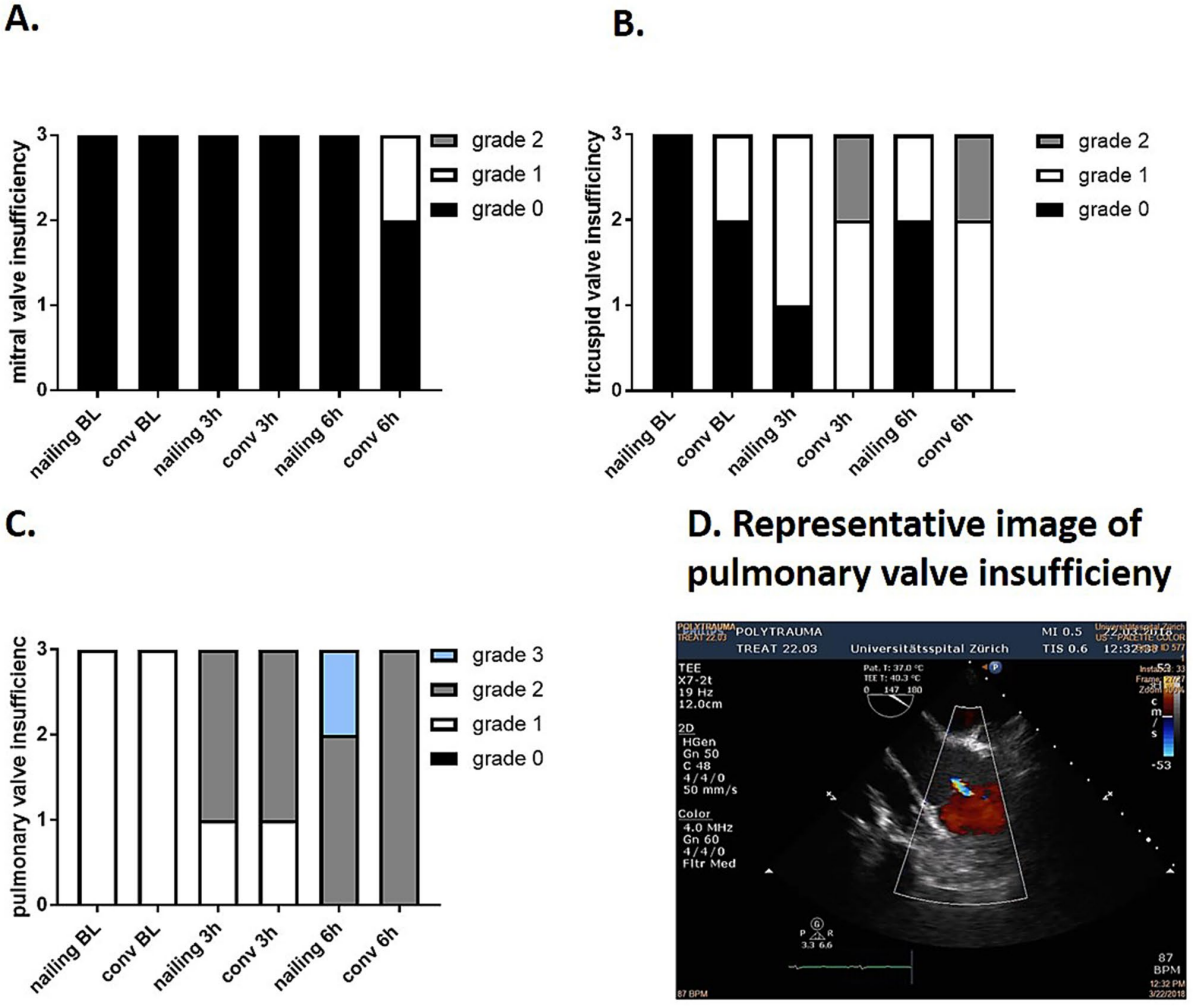
Heart valve insufficiency. (**A**) Subgroup analysis of mitral valve insufficiency at baseline (BL), 3 h and 6 h after multiple trauma (femur fracture, liver laceration, chest trauma and hemorrhagic shock) in pigs with either femoral nailing or conventional reaming as therapeutic treatment. (**B**) Subgroup analysis of tricuspid valve insufficiency in at BL, 3 and 6 h after trauma. (**C**) Subgroup analysis of pulmonary valve insufficiency determined at BL, 3 h and 6 h after trauma after femoral nailing and conventional reaming detected by transesophageal echocardiography. (**D**) Representative image of pulmonary valve insufficiency. Femoral nailing group n = 3, conventional reaming n = 3.

Furthermore, we assessed the functionality of heart valves to detect potential insufficiencies or stenoses. The aortic valve did not show any functional impairment in the multiple trauma groups (Data not shown). The mitral valve presented an insufficiency grade 1 in 20% of the animals with conventional reaming 6 h after trauma (Fig. 4A). Furthermore, the tricuspid valve was impaired in 5/6 animals after trauma (Fig. 4B). 6 h after trauma the tricuspid valve was sustained impaired in 4/6 animals (Fig. 4B). Interestingly, a pulmonary valve insufficiency grade 1 was found in most of the multiple trauma animals at baseline. During the observation period, we have seen an aggravation of the pulmonary valve insufficiency. 6 h after multiple trauma 6/6 of the animals presented a grade 2 or 3 pulmonary insufficiency. (Fig. 4C). In Fig. 4D a representative image of pulmonary valve impairment measured by echocardiography is shown (Fig. 4D).

**Figure 5:**
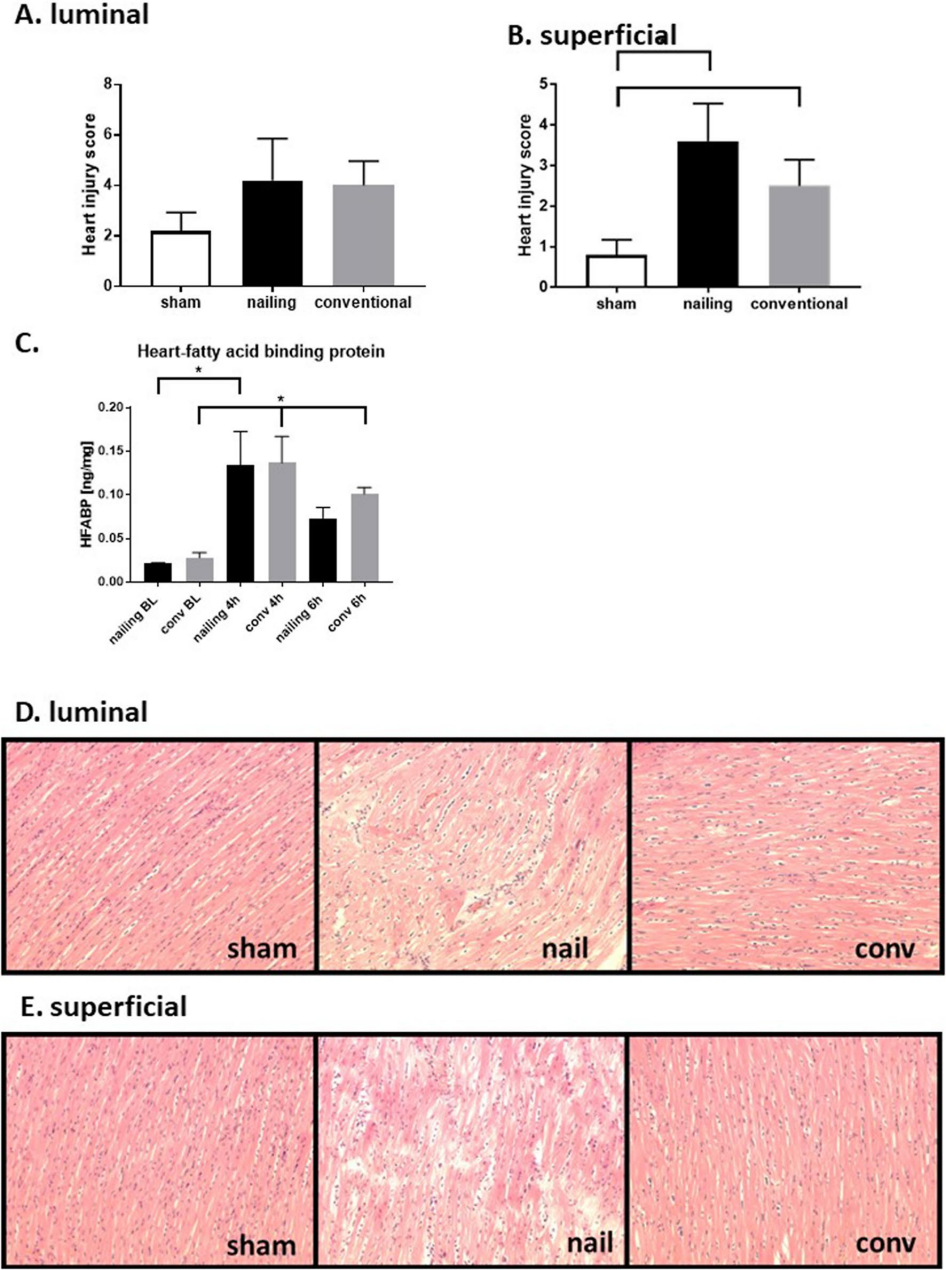
Cardiac damage after multiple trauma in pigs measured by echocardiography. (**A**,**B**) Heart injury score reflecting histomorphological sections of H.E. stained sections of the left ventricle 6 h after trauma, scored for apoptosis, contraction band necrosis, neutrophilic infiltration, intramuscular bleeding, rupture, edema and ischemia, *p < 0.05, n = 5 in each group compared to sham treated animals. (**C**) Heart-fatty acid binding protein (HFABP) in ng/ml detected in both subgroups at BL, 4 h and 6 h after multiple trauma. (**D**) Representative images of H.E: stained sections from the luminal left ventricle. (**E**) Representative Images of the superficial issue layers stained by H.E. *p < 0.05 compared to baseline, n = 5 in each group.

In order to determine early myocardial damage after multiple trauma in pigs, we developed a cardiac injury score to evaluate histomorphological changes in H.E. stained sections^21,22^. This score is presented in Fig. 5. There was an increase of histomorphological damage on the surface of the left ventricle (Fig. 5B), whereas an increase of the heart injury score in the luminal layer was only presented as a trend (Fig. 5C). In accordance with this observation we found a significant increase of the heart specific damage marker HFABP (Fig. 5C). Therefore, we assume cardiac injury after multiple trauma with haemorrhagic shock.

## Discussion

For the first time to our knowledge, we conducted a detailed analysis of EMD by TOE, diverse blood pressure parameters and valvular function after multiple trauma in pigs. Our analysis of heart rate and blood pressure revealed a reduction of blood pressure while heart rate was increased reactively during haemorrhagic shock period. The shock index (SI), which was calculated as SI = HR/systolic blood pressure, is often described in literature as a valid predictor for mortality, as a useful tool for management of triage of patients with multiple trauma. Furthermore, SI has been described to correlate with the length of intensive care unit (ICU) stay, the days of invasive ventilation, the need for transfusion and the development of septic complications in humans^23–26^. Our results presented in Fig. 1 were in accordance with these manifold investigations of blood pressure and heart rate after trauma with a haemorrhagic component. Hence, our parameters seem valid criteria to evaluate the invasiveness of our multiple trauma model.

Systemic analysis of cardiac damage markers revealed an increase of HFABP which is known to be a very early marker for cardiac damage after myocardial infarction and after trauma^10^. Especially, in the combination with ECHO measurements, the systemic increase of cardiac damage markers such as HFABP demonstrates EMD after trauma.

The present results are based on a broad analysis of cardiac functional impairment after trauma. One important consequence of cardiac injury, especially in case of direct mechanical impact on the chest, is the impairment of heart valves. The literature describes that traumatic rupture of the chordae tendineae leads to acute severe mitral regurgitation, in case of motor vehicle accidents^27^. Furthermore, different case reports described trauma associated with severe tricuspid regurgitation^28,29^. However, published case reports of traumatic valvular damage vary widely on observed symptoms: patients are described to be asymptomatic for years or become hemodynamic instable directly after trauma. Most of the reported patients developed symptoms within the first 7 days^30,31^. Valvular lesions resulted from high-energetic direct trauma on the chest such as car accidents or falls from great heights. The most likely mechanism is the sudden deceleration or compression of the blood column in the heart during the vulnerable phase of the cardiac cycle^30^. Pre-existing valvular diseases are associated with an increased risk of developing a valvular disorders after blunt chest trauma and penetrating thoracic trauma^32–35^. The most susceptible valves seem to be the atrioventricular ones^20,36^. Frequently in case of traumatic valvular dysfunction, there is a minimal initial valvular regurgitation, that might progress gradually, until cardiovascular surgery is necessary^1,37^. In contrast to atrioventricular valves, aortic valve regurgitation results from sudden increase in intrathoracic pressure against the closed valve and is associated in multiple trauma patients with a sternum or multiple rib fractures^20,38,39^. In contrast to the literature, in the present model of multiple trauma there was no evidence of any aortic valve participation. Traumatic valvular lesions and their consequences might be currently underestimated due to a wide variation of symptoms and the considerable diagnostic effort. In the present study, we observed mitral valve insufficiency in the pigs 6 h after multiple trauma and conventional reaming (Fig. 4A). Mitral valve injury typically manifested as pulmonary edema and hypotension^40^. Furthermore, we reported a high incidence of trauma-induced tricuspid impairment in Fig. 4B.

Tricuspid valve regurgitation was described as the most common cardiac complication after blunt chest trauma^41^, with a rising incidence in the last decade, caused by improved diagnostic tools^42^. The common lesion is a subvalvular rupture of the anterior papillary muscle^43^. Causes of delayed tricuspid regurgitation are papillary muscle contusion (with hemorrhage), inflammation or necrosis^44^. Therefore, in future studies of chest trauma-related cardiac injuries papillary muscle biopsies should be taken into consideration.

Next to the alterations in valvular function after multiple trauma, we further observed changes in the diastolic function of the heart. Diastolic dysfunction can occur isolated without a compromise of the systolic function, which is called heart failure with preserved ejection fraction (HFpEF). Diastolic parameters, which are accessible by cardiac catheterization and echocardiography, are the left ventricular end diastolic pressure (LVEDP) and volume (LVEDV). It is considered as an important measurement of ventricular compliance, intravascular volume as well as pressure and further could help to identify patients with an increased risk of a clinical manifestation of heart failure^45^. This value is elevated (> 15 mmHg) in patients with coronary heart disease or myocardial ischemia^45,46^. Diastolic function measured by increased LVEDP is a parameter of asynchronous myocardial relaxation and therefore of cardiac stiffness^46^. In the present study, we observed a reduction of the LVEDP in the multiple trauma group compared to baseline (Fig. 2C). The fact that LVEDP decreased can be the result of hypovolemia as demonstrated by decreased LVEDV. Another factor that might point to this is the decrease of the diastolic blood pressure as presented in Fig. 1F. Furthermore, general decrease of LV pre-load caused by fusion of tricuspid and pulmonary valve regurgitation. Another parameter of diastolic function is the ratio between E and A wave as measured by TEE among the mitral valve. As presented in Fig. 3E, no changes in the E/A ratio occurred in the pigs with multiple trauma in the present observation period. We measured an increase of the mitral valve deceleration time (Fig. 3C), which is also associated with diastolic dysfunction in the literature. The E/A ratio is described to depend heavily on multiple interrelated factors and is therefore not useful for detect diastolic function without taking other parameters into account^46^. Diastolic dysfunction was shown to be promoted by cardiac macrophages and their increased production and release of IL-10^47^. An increase of cardiac macrophages after chest trauma has been detected previously^48^.

In the present multiple trauma model, we failed to detect changes in cardiac output or ejection fraction in any of the investigated groups 6 h after multiple trauma. These results are in contrast to earlier transthoracic measurements after multiple trauma in pigs^10^. Measurement of cardiac output especially in hypovolemic conditions is time consuming and depends on the investigators experience^15^. Therefore, cardiac output does not seem to be the ideal parameter for cardiac function assessment after trauma. One limitation of the study is that it is rather difficult to ensure the use of TOE as a diagnostic procedure in multiple trauma in the emergency room (ER). Here, transthoracic echocardiography or ECG triggered CT are the more realistic methods to evaluate cardiac function. In the clinical setting TOE is more often used in order to access cardiac function additionally in anesthetized patients in the OR or for follow-up measurements on the intensive care unit.

In patients with previously diagnosed hypertension and left ventricular hypertrophy, traumatic bleeding can lead to dynamic obstruction of the left ventricular outflow tract^15^. In the present study we detected a reduction of the LVOT VTI 6 h after multiple trauma in pigs (Fig. 3F). A reduction of the LVOT VTI has been described as a predictor of heart failure and increased mortality^49^. LVOT VTI is a sensitive systolic marker low cardiac output, cardiogenic shock and impaired ability to keep up the systemic tissue perfusion and metabolic demands^49^. Additionally, a hyperdynamic left ventricle might also be a sign of traumatic hypovolemia. This condition is well-reported in cases of low peripheral resistance after sepsis or as expression of post-traumatic inflammation^50^. Furthermore, traumatic lesions far away from the thorax were to influence the systolic function of the heart: 22% of patients after a traumatic brain injury (TBI) developed systolic dysfunction, caused by a maladaptive catecholamine excess state^51^.

## Conclusion

Taken together, this study observed complex EMD and valvular impairment after multiple trauma in pigs as accessed by TOE. These results were accompanied by systemic release of the cardiac damage marker HFABP. Translating these findings into clinical medicine, detection of EMD and progressive valvular dysfunction might be crucial and therapeutically relevant and therefore may improve treatment of patients after multiple trauma.

## Materials and methods

### Animals

This study is a part of a large porcine multiple trauma project, which was conducted by the TREAT research consortium. This model and the animal experiments were described previously by Lackner et al.^52^.

The animal housing and experimental protocols were approved by the Cantonal Veterinary Department, Zurich, Switzerland, under the licence number: ZH 138/2017 (approval for the study was given to the Department of Trauma, University Hospital of Zurich, Zurich, Switzerland). This study was conducted in in accordance with the Swiss Animal Protection Law. Housing and experimental procedures also conformed to European Directive 2010/63/EU of the European Parliament and of the Council on the Protection of Animals used for scientific purpose and to the Guide for the Care and Use of Laboratory Animals (Institute of Laboratory Animal Resources, National Research Council, National Academy of Sciences, 2011). We included 15 male pigs (Sus scrofa domestica) in the present study. The animals had a mean body length of 123.6 cm and a mean weighting of 50 ± 5 kg. Pigs underwent either multiple trauma (n = 10) or sham-procedure (n = 5). The pig model was described previously by Horst et al*. *^53^*.* The animals received a premedication with ketamine (20 mg/kg body weight), azaperone (1–2 mg/kg body weight) and atropine (0.1.0.2 mg/kg body weight) by intramuscular injection. Furthermore, the continuous anesthesia was performed by propofol infusion (1–2 mg/kg body weight). The anesthesia was maintained during the observation period of 6 h by continuous propofol application (5–10 mg/kg/h) furthermore as pain medication sufentanyl was given as perfusion over the whole experimental period (1 µg/kg/h).

### Multiple trauma in pigs

The conducted, well-standardized multiple trauma model is defined by a penetrating chest trauma, a liver laceration, a haemorrhagic shock and a femur fracture. All in All, an Injury severity score (ISS) > 16 was guaranteed. Femur fracture was induced by a bolt gun (Blitz-Kernen, turbocut JOBB GmbH, Germany) loaded with cattle-killing cartridges (9 × 17; DynamitNobel AG, Troisdorf, Germany). The chest trauma was also induced by the shock wave of the bolt gun. Therefore, a pair of panels (0.8 cm, lead 1.0 cm thickness) was placed on the right dorsal lower chest as described previously^53,54^. The abdominal trauma was simulated by a penetrating hepatic injury, which was induced by cross-like incision halfway through the liver tissue. After a short period of uncontrolled bleeding (30 s), liver package was performed. The multiple trauma was completed by a pressure-controlled haemorrhagic shock (MAP of 25 ± 5 mmHg, max. 45% of total blood volume). After 60 min of shock, animals were resuscitated according to established trauma guidelines (ATLS, AWMF-S3 guideline on Treatment of Patients with Severe and Multiple Injuries) by adjusting FiO_2_ and infusing additional fluids (Ringerfundin, 2 ml/kg body weight/h).

Sham procedure (n = 5) included instrumentation and anaesthesia without any trauma. We randomized the multiple trauma group in two therapy arms: pigs received either femoral nailing without reaming (n = 5) or standard reaming (n = 5). In both groups a shortened conventional tibia nail was introduced. The conventional reaming is conducted by a standard drill without irrigation and suction of the intramedullary contents. The present study contains data from both treatment groups summarized as a multiple trauma group, as well as subgroup analysis.

Hemodynamic parameters were continuously monitored for 6 h (DataScience International Ponemah V5.1 New Brighton, MN, US). At the end of the experiment, the animals were euthanized under deep, general anaesthesia by intravenous Na-Pentobarbital overdose. We collected serum and plasma samples at the following timepoints: baseline, 4 h and 6 h after trauma. After centrifugation (1500*g* for 12 min at 4 °C), serum and EDTA-plasma were removed and stored at − 80 °C. Tissue samples of the left ventricle were obtained 6 h after trauma. To ensure a detailed analysis, we separated the superficial and the luminal layers of the ventricle and fixed the tissue with 4% formalin, followed by embedding in paraffin. Moreover, tissue was quick-frozen in liquid nitrogen to further analyse gene expression by PCR. This animal experiment was also the base of the study, described by Lackner et al.^52^.

### Transesophageal echocardiography (TOE) in pigs

Imaging was performed according to the recommendations using a standard cardiac ultrasound machine (Cx50 xMATRIX, Phillips Healthcare, Germany with the X7-2t probe and the S5-1 ultrasound probe for additional transthoracic measurements). Serial imaging was performed before trauma, 3 and 6 h after trauma by an experienced investigator for echocardiography in pigs. The following parameters were measured: cardiac output (CO) in l/min, the ratio (A/E) of peak velocity blood flow in early diastole (the E wave) to peak velocity flow in late diastole caused by atrial contraction (A wave), mitral deceleration time (MV desc time) in s, left ventricular outflow tract velocity time integral (LVOT VTI) in cm, right ventricular outflow tract velocity time integral (RVOT VTI) in cm, intraventricular septum thickness in end systole (IVSs) and end diastole (IVSd) in cm, right ventricular outflow tract diameter (RVOT diameter) in cm and left ventricular outflow tract diameter (LVOT diameter) in cm. Additionally, an experienced investigator evaluated the heart valve function by means of color Doppler echocardiography imaging. The observed insufficiencies were rated from grade 0 (no insufficiency) to grade 3 (massive insufficiency). We measured insufficiencies in the aortic, the pulmonary, mitral and tricuspid valve at Baseline, 3 h and 6 h after trauma.

In addition, we analyzed the continuously measured arterial blood pressure curves over 6 h and detected the following parameters: heart rate (HR) in beats per minute (bpm) (actually: pulse wave), arterial systolic and diastolic blood pressure and MAP in mmHg at baseline, trauma, 1, 2, 3, 4 and 6 h after trauma. Furthermore, at specific timepoints (BL, 3 h and 6 h) the following parameters were measured: left ventricular end-diastolic pressure (LVEDP) in mmHg, maximal left ventricular/systolic pressure (LVP max) in mmHg, minimal left ventricular/diastolic pressure (LVP min) in mmHg, contractility index (CI) in l/min/m^2^, maximum positive value of the first derivative of the pressure that occurs during cardiac cycle (+ dp/dt max) in mmHg/s, maximum negative value of the first derivative of the pressure that occurs during the cardiac cycle (-dp/dt min) in mmHg/s and tension-time index (TTI = integration between the LVEDP point and -dp/dt max) by placing a pig tail catheter 5Fr.) in the left ventricle as previously published^55^, the shock index during the observation period was calculated as heart rate/systolic pressure.

### Heart-fatty acid binding protein (HFABP)-ELISA in pigs

Plasma samples were analyzed by using ELISA kits according to the manufacturer instructions. Heart fatty acid binding protein concentrations were detected by a pig cardiac fatty acid binding protein ELISA kit (life diagnostics, West Chester, PA, USA). We decided to use HFABP for systemic cardiac damage analysis instead of troponin, since HFABP is known to increase early after cardiac contusion.

### Heart injury score

To evaluate the cardiac damage a fast H.E. staining-kit (Morphisto, Frankfurt am Main, Germany) was used to stain the left ventricle 6 h after trauma. To evaluate histomorphological changes, H.E. stained sections were scored for (1) apoptosis, (2) contraction band necrosis, (3) neutrophilic infiltration, (4) intramuscular bleeding, (5) rupture, (6) edema and (7) ischemia. This score was previously established by our working group^21,22^. For each animal, 4 fields of vision (magnification: 100 ×) were summarized in one mean score per section.

### Statistical procedures

Mean values were analysed by one-way ANOVA followed Dunnett’s or Tukey’s multiple comparison test. Therefore, changes were defined as statistically significant with a p ≤ 0.05. Statistical analysis and graphical presentation were conducted by using the GraphPad Prism 7.0 software (GraphPad Software, Incorporated, San Diego, CA, USA). The values were presented as mean ± SEM in all graphs.

## Supplementary information

Supplementary Figure.

## Abbreviations

ATLS: Advanced trauma life support
A-wave: Peak velocity flow in late diastole
BL: Baseline
CI: Contractility index
CO: Cardiac output
Diast.: Diastolic blood pressure
+ dp/dt max: Max positive value of first derivate of pressure during cardiac cycle
-dp/dt min: Max negative value of the first derivate of pressure during cardiac cycle
ECHO: Echocardiography
EDV: End-diastolic volume
EF: Ejection fraction
ELISA: Enzyme-linked immunosorbent assay
EMD: Early myocardial damage
ESV: End-systolic volume
E-wave: Peak velocity blood flow in early diastole
FELASA: Federation of European laboratory animal science association
FiO_2_: Fraction of inspired oxygen
GV-SOLADES: Society of laboratory animal science
HFABP: Heart-type fatty acid binding protein
HFpEF: Heart failure with preserved ejection fraction
HR: Heart rate
H.E.: Haematoxylin and eosin
IL: Interleukin
ISS: Injury severity score
IVss/d: Intraventricular septal end systole/diastole
LVEDP: Left ventricular end diastolic pressure
LVOT/ RVOT VTI: Left/right ventricular outflow tract velocity time integral
LVP max/min: Max/min left ventricular pressure
MAP: Mean arterial pressure
MV desc time: Mitral desceleration time
SEM: Standard error of the mean
Systole.: Systolic blood pressure
TBI: Traumatic brain injury
TNF: Tumor necrosis factor
TnI: Troponin I
TOE: Transesophageal echocardiography
TTI: Tension-time index
