# Supplementary figures and images for: Early myocardial damage (EMD) and valvular insufficiency result in impaired cardiac function after multiple trauma in pigs
Source: Sci Rep. 2021 Jan 13;11:1151. doi: 10.1038/s41598-020-80409-8 (PMC7806767; doi:10.1038/s41598-020-80409-8)

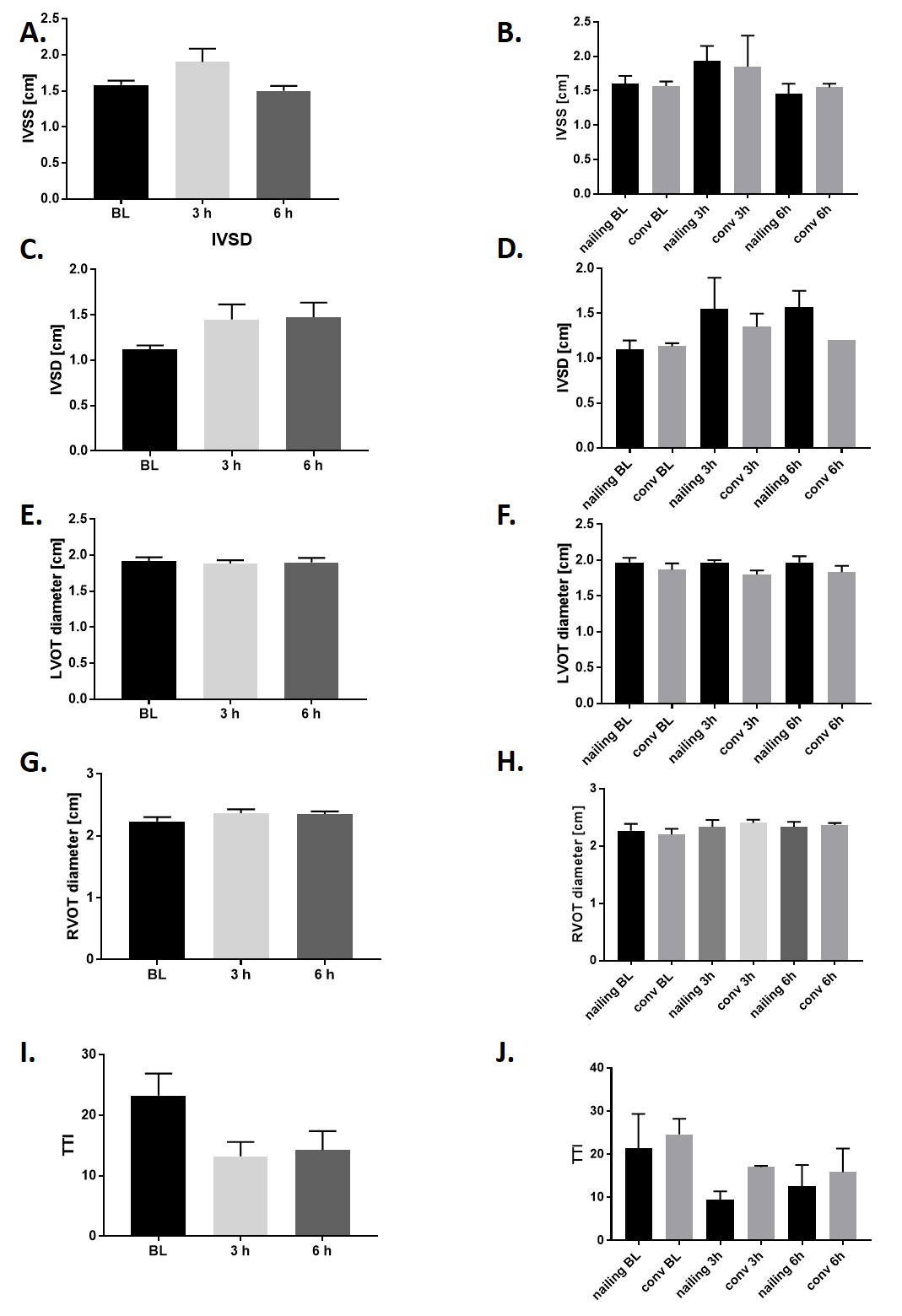

Supplement: Supplementary file 1 — Supplementary Figure. [file 41598_2020_80409_MOESM1_ESM.jpg]
